# Changes in the availability and affordability of subsidised artemisinin combination therapy in the private drug retail sector in rural Ghana: before and after the introduction of the AMFm subsidy

**DOI:** 10.1093/inthealth/ihw041

**Published:** 2016-11-16

**Authors:** Evelyn K. Ansah, Christopher JM Whitty, Constance Bart-Plange, Margaret Gyapong

**Affiliations:** a Dangme West District Health Directorate, Ghana Health Service, P.O. Box DD1, Dodowa, Ghana; b London School of Hygiene & Tropical Medicine, Keppel Street, London WC1E 7HT, UK; c National Malaria Control Programme, Ghana Health Service, P.O. Box KB493, Korle-Bu, Accra, Ghana; d Dodowa Health Research Center, Ghana Health Service, P.O. Box DD1, Dodowa, Ghana

**Keywords:** Affordability, AMFm, Chemical Seller, Drug Retail Shop, Ghana, Malaria

## Abstract

**Background:**

Most people with febrile illness are treated in the private drug retail sector. Ghana was among nine countries piloting the Global Fund Affordable Medicines Facility – malaria (AMFm). AMFm aimed to: increase artemisinin combination therapy (ACT) affordability; increase ACT availability; increase ACT use; and ‘crowd out’ artemisinin monotherapies.

**Methods:**

Three censuses were carried out 2 months before (2010), 2 months after and 2.5 years after (2013) the first co-paid ACTs to assess changes in antimalarial (AM) availability and price in private retail shops in a Ghanaian rural district to assess the sustainability of the initial gains. Supply, stock-out and cost were explored.

**Results:**

Of 62 shops in the district, 56 participated with 398, 388 and 442 brands of AMs in the shops during the 3 censuses. The proportion of ACTs increased over the period while monotherapies reduced. Herbal-based AM preparations comprised 40–45% of AMs in stock with minimal variation over the period. ACTs were the most sold AM type for all ages but overall buying and selling prices of Quality Assured-ACTs increased by 40–100%.

**Conclusions:**

Initial gains in ACT availability were sustained, but not improved on 2.5 years after AMFm. Widespread availability of unproven herbal medicines is a concern; AMFm had little impact on this.

## Introduction

In common with much of Africa, the majority of adults and children with febrile illness in Ghana, including the poorest, are treated in the private retail sector.^1–3^ Accepting this reality, and on advice from many economic commentators that subsidies would be needed, the Global Fund introduced the innovative Affordable Medicines Facility – malaria (AMFm) initiative, which subsidised high-quality Artemisinin Combination Therapy (ACT) drugs to try to ensure that those buying from the sector had access to effective combinations.^4^ The four main objectives of AMFm were to: increase ACT affordability; increase ACT availability; increase ACT use, including among vulnerable groups; and ‘crowd out’ oral artemisinin monotherapies. AMFm was considered audacious both in its aims and scope, and was not without controversy, with some commentators very skeptical that an intervention in the private sector was wise. Initial trial and evaluation evidence was however broadly supportive.^5–8^

Ghana was among the nine countries that piloted the first phase of the strategy. Ghana was also the first country to receive the subsidised ACTs (called ‘co-paid’), and availability of ACTs increased from 31% to 83% nationwide. The price of ACTs dropped from about US$7 to US$0.75 for adults and from US$5 to US$0.5 for children by December 2011 according to initial assessments.^9^ Experience elsewhere suggested a mixed picture; for example in Tanzania, although availability was widespread, there were variations across districts not based on remoteness and average prices fell from US$1.03 to US$0.81 over the study period, even though the government recommended retail price for the subsidised ACTs was US$0.64.^10^ In Nigeria and Ghana, Bate et al found a few months after introduction of the subsidised ACTS that they were lower in price than many of the other drugs collected, but by less than anticipated or stipulated by the participating governments.^11^

Initial assessments were broadly positive, but initial assessments undertaken after a major change often do not reflect subsequent operational reality. The system can either revert back towards the previous norm, or initial successes can be built on. This study aimed to look at the effect in a typical Ghanaian district over the change period, and then once the change had become established practice. Chemical shops, the principal private retail outlet for drugs in many remote areas were the focus of the study. The study assessed changes in ACT availability in the private retail shops at three different time periods; 2 months before, 2 months after and 2.5 years after the first co-paid ACTs arrived in Ghana in August 2010. We also assessed prices of antimalarials (AM) in the shops 2.5 years after AMFm in a rural district in Ghana with an original fixed co-paid ACT price of 1.50 Ghana cedis(ghs) (USD1.00), and the effect on availability of artemisinin monotherapy and other antimalarial drugs in the private drug retail shops.

## Materials and methods

### Study site and population

The study was carried out in the Dangme West District of Ghana, a rural and deprived district with an estimated 2012 mid-year population of 130 570 based on the 2010 census and recently divided into two separate administrative districts.^12^ Most of the population lives in scattered small communities of less than 2000 people. Vehicular transport is unavailable in many parts of the district and people have to walk long distances (approx. 2–6 h) to reach the nearest main road making access to formal care difficult. The district is typical of poor disadvantaged rural and semi-rural districts across the country. Poverty is widespread. Health services are delivered from one district hospital, four health centres, 13 Community Health Planning and Services (CHPS) compounds and five private health facilities. CHPS compounds are the lowest level of service delivery in the health system, where services are delivered by community health nurses (auxiliary nurses). The district hospital and three of the health centres are located in the four largest towns in the district. A total of 56 chemical sellers and 6 pharmacies also sell pharmaceutical products and their diagnostic practice is described elsewhere.^12^ Chemical sellers are regulated by law and supervised by the Pharmacy Council, which also regulates pharmacies. To be eligible to apply for a chemical seller's license, a person must possess a minimum qualification of General Certificate of Examination (GCE) Ordinary Level Certificate or a Senior Secondary School (SSS) Certificate with basic knowledge in healthcare delivery being an advantage. Applicants attend a pre-licensing training organised by the council and the licenses are renewed annually. The shops are by law allowed to supply by retail, over-the-counter medicines to members of the public in communities considered to be deprived by the Council. The authorised medicines do not generally include antibiotics but do include analgesics and antimalarials. The only antibiotic recently included is co-trimoxazole. They obtain their medicines from the same drug companies who supply medicines to pharmacies.

Earlier studies carried out in the district showed that for presumed ‘malaria’ in the household, the first action taken in order of the most common were home treatment, chemical seller, health centre, hospital, drug peddler and traditional healer in that order.^13^ Recent demographic surveillance data from the district indicated that about 67% of all deaths took place at home.^14^

### Study design

The study involved carrying out three censuses in chemical shops. The census involved the documentation of all the different types of AM in the shops as well as the retail prices. The censuses were carried out in 53 chemical shops and 3 pharmacies, out of the total of 62 operating in the district. The selected shops were also participating in an on-going trial. The first census took place 2 months before the AMFm strategy while the remaining two, took place 2 months and 2.5 years after the strategy had been implemented (Table 1).

**Table 1.** Timeline for data collection in relation to implementation of AMFm strategy in Ghana

Date	Activity
June 2010	First Census (Census 1) is carried out
August 2010	The first co-paid ACTs delivered to Ghana as part of the AMFm Strategy
October 2010	Second Census (Census 2) is carried out
April 2013	Final Census (Census 3) is carried out

ACT: artemisinin combination therapy; AMFm: Affordable Medicines Facility – malaria

At the time of the last census, a short survey was carried out in the shops to find out from the shop owners or regular attendants about the source and availability of whichever ACTs they had available, client preferences for AM and stock-out issues. A short questionnaire was administered to whoever was selling in the shop on the day of visit to the shop.

### Statistical methods

All data were checked for completeness and consistency after which they were double entered into EPI Data version 3.10 (EpiData Association, Odense, Denmark) followed by validation of the data entered. Discrepancies were resolved by retrieving the hard copy of the data collection instruments. Data cleaning and analysis were completed using Stata version 12.0 (Stata Corp, College Station, TX, USA). Statistical comparisons were considered significant at the 0.05 level. Analysis of the data involved the calculation of simple frequencies and proportions with exact confidence intervals (CIs).

### Ethical considerations

Ethical approval was obtained from the Ethics Review Committees of the Ghana Health Service (GHS) in Ghana and the London School of Hygiene & Tropical Medicine. Permission was sought from the Dangme West District Health Management Team. Informed consent was sought from the owners of the chemical shops who participated in the study. All study records, including shops and health facilities, were identified by means of study IDs.

## Results

Fifty-three chemical shops and 3 pharmacies out of 62 existing shops participated in the study, which took place at different time points spanning the period June 2010 to February 2013.

Overall, there were 398, 388 and 442 different brands of AM in the shops during the 3 censuses, respectively.

### Availability of quality assured ACTs

Antimalarials were classified into four groups as follows: Quality Assured ACTs (QUAACTs), artemisinin monotherapies, non-artemisinin monotherapies, and herbal-based AM. These four categories cover the types of AM on the market. The QAACTs are WHO recommended pre-qualified ACTs, while the herbal-based ones are locally manufactured AM from herbs, which are very popular, especially in the rural areas. Availability of ACTs increased over the period, comprising 16.6%, 42.5% and 47.7% of AM in stock in all shops, respectively. Although QUAACTs were not found in any shop during the first census they comprised 80.0% (124/155) and 87.2% (184/211) of all ACTs during the second and third censuses, respectively. An increase was seen in the proportion of all AMs that were ACTs, over time. Data are summarised in Table 2.

**Table 2.** Availability of various classifications of antimalarials in chemical shops in the Dangme West District at different time periods

Indicators	Time periods		
	Census 1 (2 months pre-AMFm) n (%)	Census 2 (2 months post AMFm) n (%)	Census 3 (2.5 years post AMFm) n (%)
Number of antimalarial brands in the shops	398	388	442
ACTs	66 (16.6%)	155 (39.9%)	211 (47.7%)
QUAACTs	NA	124(32.0%)	184 (41.6%)
Artemisinin monotherapies	38 (9.5%)	18 (4.6%)	15 (3.4%)
Non-artemisinin therapies	136 (34.2%)	27 (6.9%)	42 (9.5%)
Herbal-based antimalarials	158 (39.7%)	178 (45.9%)	(174) 39.4%

ACT: artemisinin combination therapy; AMFm: Affordable Medicines Facility – malaria; NA: not applicable; QAACT: quality assured artemisinin combination therapy

**Figure 1.**
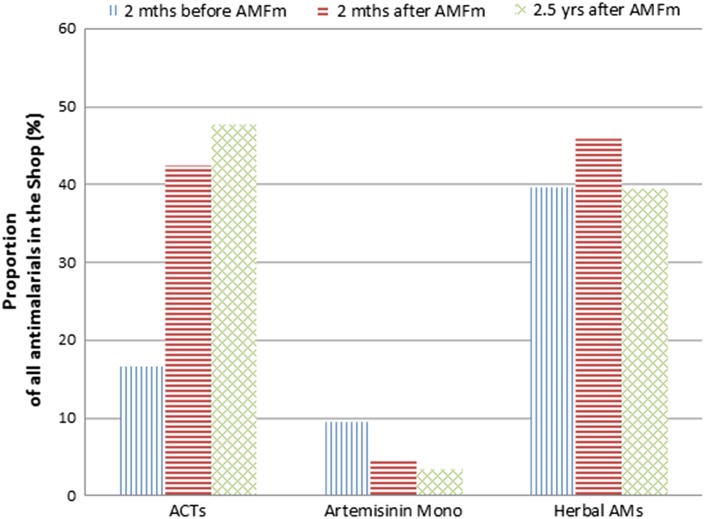
Changes in proportions of types of antimalarials found in chemical shops at three different time periods (June 2010, October 2010 and April 2013). ACT: artemisinin combination therapy; AM: antimalarial; AMFm: Affordable Medicines Facility - malaria. This figure is available in black and white in print and in color at International Health online.

The proportion of non-artemesinin therapies of all the AM in stock initially dropped sharply from 34.2% (136/398) to 6.9% (27/388) by the second census. By the third and most recent census, the proportion had increased back slightly to 9.5% (42/442). Artemisinin monotherapies comprised 9.5% (38/398), 4.6% (18/388) and 3.4% (15/442) of all AM available in the three time periods, suggesting that this change was sustained. Stocks of herbal-based AM preparations were relatively high, forming on average, 40–45% of all AM in stock in the shops. This did not change substantially over the period, constituting 39.7% (158/398), 45.9% (178/388) and 39.4% (174/442) of AM during the three censuses, respectively, so were the same before and 2.5 years after AMFm was started (Figure 1).

At the time of the survey coinciding with the last census, out of the 442 different brands of AM found in the 56 shops, 39.4% (174/442) were herbal preparations as compared to 47.7% (211/442) ACTs. QUAACTs were the most sold type of AM for both children and adults. The most sold AM for adults in the 2 months preceding the survey were said by 15 of the 56 shops to be artesunate-amodiaquine (26.8%) and artemether-lumefantrine (26.8%), respectively. For children, however, artesunate-amodiaquine was mentioned by 18 shops (32.1%) as compared to artemether-lumefantrine by 10 (17.9%) out of the 56 shops. Overall, 66.1% (37/56) of the shops said they had QUAACTs in stock on the day of the survey. Most of the QUAACTs available were artemether-lumefantrine (59.5% of all QUAACTs). Overall, 55.4% (31/56) of shops admitted to having experienced stock-outs of QUAACTs in the preceding 2 months; most of them (12/31; 38.7%) for a 1–2 week period. Only 16 of the 56 shops (28.6%) had not experienced any stock outs of the QUAACTs. Buying and selling prices of QAACTs had increased by 40–100% and shopkeepers attributed this mainly to the scarcity of the commodity and also to some degree, distributors regulating how much they sold to any one shop (Table 3).

**Table 3.** Price of various classifications of antimalarials in chemical shops in the Dangme West District at different time periods

Indicators	Costs		
	Census 1 (2 months pre-AMFm) Average cost [range] in Ghana cedis	Census 2 (2 months post AMFm) Average cost [range] in Ghana cedis	Census 3 (2.5 years post AMFm) Avg cost [range] in Ghana cedis
Number of antimalarial brands in the shops	398	388	442
QAACTs	NA	2.87 [1.00–3.00]	3.85 [1.50–7.00]
Other ACTs	4.61 [0.50–13.00]	4.92 [1.5–15.00]	5.56 [1.00–15.00]
Artemisinin monotherapies	3.04 [2.50–6.00]	3.16 [1.20–3.80]	3.66 [3.00–5.00]
Non-artemisinin therapies	1.02 [1.00–13.50]	1.59 [0.30–5.50]	1.45 [0.50–4.00]
Herbal-based antimalarials	2.14 [1.50–5.00]	4.35 [3.00–7.00]	4.52 [2.40–8.00]

ACT: artemisinin combination therapy; AMFm: Affordable Medicines Facility – malaria; NA: not applicable; QAACT: quality assured artemisinin combination therapy

### Prices of antimalarials

Generally, adult AM were bought and sold at higher prices at all times. When the AMFm medicines first arrived, shop attendants bought the QUAACTs for children at a mean price of 0.79 ghs (US$0.53) and sold them at 1.26ghs (US$0.84). For adults they bought and sold them at 1.05ghs (0.70USD) and 1.61 ghs (1.07USD), respectively. However, 2.5 years later they were buying the children's doses at 1.20 ghs (US$0.80) and selling them at 1.78ghs (US$1.19) while those of adults were being bought and sold at mean prices of 1.65ghs (US$1.10) and 2.29 ghs (US$1.53), respectively. The shop attendants said they sometimes were only able to obtain the AMFm medicines if they purchased other slow moving medicines since the distributors carried out conditional sales. This contributed to the price of the AM going up in order to cover the loss they would make on the cost of the other medicine; some which were likely to expire.

Percentage mark-up on adult QUAACTs were lower 2.5 years after the AMFm was introduced as compared to the time the medicines first arrived (41.3% vs 54.0%). This may have been due to the fact that the prices at which shopkeepers bought the QUAACTs had gone up considerably over time and they did not feel able to increase the prices of the medicines to the extent that would provide them with the same mark up as before. For children, the change in percentage mark-up was minimal (49.4% vs 58.2%).

## Discussion

Some countries are considering putting some of their funds from the Global Fund into the private sector as this is where many patients with malaria go.^15^ AMFm was the first major attempt to do this for malaria on a widespread basis. Of the four main objectives of AMFm, in this area of Ghana during this study there was reasonable success in two: to increase ACT availability in the private sector, although stock outs remained a major problem; and to ‘crowd out’ oral artemisinin monotherapies. In this area of Ghana there was only a slight increase in affordability. Initial gains in these domains were broadly sustained over the subsequent 2.5 years, but not improved upon. Stocking of untested herbal products, which are heavily promoted through the media for malaria, was unchanged.

Ghana is a good place to test AMFm concepts of subsidy of key commodities in the private sector, as it has a relatively well regulated public sector but also a thriving private sector where many patients go. Early studies suggested that the subsidy might not reach rural areas - here, as in some other sites, it did.^16,17^ Several previous assessments of AMFm have either only looked at the first year after implementation, which can examine initial effect but not sustained effect, or used cross-sectional studies at a single point in time.^5,18^

A decision by the Global Fund to incorporate AMFm into regular funding over the 2012–13 period (so countries have to trade off funding for public sector support and private sector subsidy) means the mechanism has now changed significantly, but the principles of subsidy in the private sector remain the same.

The issue of stock outs (55% of shops in the preceding 2 months in the last time period) was however a serious one, and had an impact on both availability and price. It is probably the reason why prices did not drop further. The last time period is the most interesting one as it represents the new normal state after initial euphoria and inevitable startup problems. The fact that stock outs were still occurring at this rate over 2 years later cannot be ascribed to initial teething problems. In their Tanzanian study, Cohen and others found that promotions and advertisements on the radio increased awareness of ACTs, and possibly increased desirability for the shopkeepers to stock them.^16^ Improvements in communication can help raise awareness of AMFm products, and could be used to counter the use of untested herbal products, but in the absence of secure drug supply this will only have limited impact.^19^ This is especially important as the herbal-based AM are freely advertised on radio in this setting and for many people, especially in the rural areas, these would be the AM they would know by name. Client preferences may influence which AM shopkeepers stock most^16^ and this may explain why in this setting, herbal-based AM constituted, on average, 40% of all AM stocked.

The study inevitably has limitations. In common with all before-and-after designs this study can only demonstrate association, not causality. The very rapid change over the AMFm period followed by little change thereafter does however suggest AMFm and associated publicity was the main cause of the change in availability of drugs, and supports the findings of initial assessments of AMFm during its introduction elsewhere in Africa.^20^ The study was able to examine three of AMFm's aims over a 2.5 year period after AMFm was introduced: to increase ACT affordability; to increase ACT availability; and to ‘crowd out’ oral artemisinins. It is not able directly to examine the final one, to increase ACT use, and there is evidence that in some settings increasing availability of affordable ACTs in the private sector does not in itself necessarily increase use, although in others it has been found to.^21,22^

### Conclusions

AMFm was initially partially successful in its aims in this area in increasing availability of ACTs and helping crowd out artemisinin monotherapy - this study shows that initial gains were sustained 2.5 years later but not improved on. In order to prevent reversal of the gains in malaria control over the last decade, consistent supply of QAACTs to the private retail sector must be assured. Stock outs (limiting supply for existing demand) may play a major role in the price of ACTs as well as reducing availability directly. The continuing popularity of unproven herbal remedies for malaria is a concern and AMFm seems to have made little difference to this in Ghana.
